# Translation, adaptation and psychometric testing of a tool for measuring nurses’ attitudes towards research in Indonesian primary health care

**DOI:** 10.1002/nop2.72

**Published:** 2016-11-06

**Authors:** Kurnia Rachmawati, Tim Schultz, Lynette Cusack

**Affiliations:** ^1^ School of NursingUniversity of Lambung MangkuratBanjarmasinIndonesia; ^2^ School of NursingThe University of AdelaideAdelaideSouth AustraliaAustralia

**Keywords:** attitudes, construct validity, developing countries, factor analysis, instrument development, nursing research, primary health care, psychometric testing

## Abstract

**Aim:**

The purpose of this study was to translate, adapt and psychometrically test the Nurses’ attitudes towards and awareness of research and development within nursing (ATRAD‐N) version II for measuring nursing research and research utilization in Indonesian primary health care nurses.

**Design:**

Cross‐sectional survey.

**Method:**

The translation process was conducted by applying the forward and back‐translation method. Adaptation and content validity was assessed by six experts in Indonesia. The psychometric testing was performed using factor analysis and Cronbach's alpha coefficient on a sample of 92 primary health care nurses in South Kalimantan, Indonesia in 2013.

**Results:**

The translated instrument showed acceptable content validity with index of .97. The factor analysis (Principal Component Analysis with Direct Oblimin rotation) obtained a five‐factor structure that differed from those identified in previous studies. The cumulative percentage of variance was 56.5%. The Cronbach's alpha coefficient for individual factors ranged from .719 ‐ .884. The resulting form of the Indonesian ATRAD‐N was found to have acceptable content validity and homogeneity reliability but not construct valid in Indonesian settings.

## 1 Introduction

Several studies have focused on assessing instruments designed to measure and assess research utilization in practice and individual factors associated with research utilization (Estabrooks & Wallin, 2004; Frasure, 2008; Squires, Adachi & Estabrooks, 2008; Squires et al., 2011). Most of these studies have been conducted in hospital settings (Nilsson Kajermo, Alinaghizadeh, Falk, Wandell & Tornkvist, 2013) and in developed countries (Squires et al., 2011). The published data on instruments designed to measure and assess research utilization in primary health care typical in developing countries are scant. Given the contextual differences between hospital and primary health care as well as developed and developing country settings, it is important to provide an instrument in this field that is valid and reliable across settings.

In this article, we describe a process of translation, adaptation and psychometric testing of an instrument namely Nurses’ attitudes towards and awareness of research and development within nursing (ATRAD‐N) version II by Bjorkstrom and Hamrin (2001) to be used in primary health care settings in Indonesia. In 2001, the originators report the development of the instrument which was based on a review of literature concerning nursing research and two previous studies conducted in Sweden. In several tests, the instrument was found to have acceptable measures of reliability and validity. In 2005, the instrument was modified and tested to focus on primary health care settings (Nilsson Kajermo et al., 2013). All of the psychometric tests of the instrument have been conducted in developed countries.

## 2 Background

Research utilization, the use of research evidence to inform practice, has become a main concern in nursing practice worldwide as the evidence‐based practice movement extends from a focus of “what is evidence and how can it be summarized” to “how can evidence be used to inform daily clinical practice” (Estabrooks, 2009; Estabrooks, Wallin & Milner, 2003b; Schneider, 2013). All nurses, even those in rural areas, should be able to use scientific evidence to guide their practice (Olade, 2004). However, research utilization is a complex process that requires synergistic efforts to be successfully implemented (Mehrdad, Salsali & Kazemnejad, 2008). Estabrooks (2009) outlined several determinants influencing research utilization: individuals, organization and innovation (Estabrooks, 2009).

Research utilization is a new concept in Indonesian nursing and research modules have only recently been added to the nursing curriculum (Indonesian National Nurses Association, 2005). There are also few publicly available data that can be used to inform nursing research and research utilization in developing countries like Indonesia (Pearson & Jordan, 2010). As a result, the implementation of evidence‐based practice is complex and difficult for Indonesian nurses. As in other developing countries, several factors contribute to this situation (Mehrdad et al., 2008; Tsai, 2000). Poor quality of education and lack of strategies to enhance the use of research findings are two common barriers in research utilization and research participation (McKenna, Ashton & Keeney, 2004; Oh, 2008; Tsai, 2000).

The primary health care system in Indonesia is the Pusat Kesehatan Masyarakat (Puskesmas) (public health centers), the functional health organization unit (Abdullah, Hort, Abidin & Amin, 2012). Puskesmas are front line health service institutions that have responsibility for providing comprehensive and integrated services to the community (Ministry of Health‐Republic of Indonesia, 2012). In collaboration with other related sectors, the centres implement national and regional health programs, including those dealing with health promotion, illness prevention, treatment of diseases and rehabilitation to all community groups (Department of Health‐Government of Indonesia, 1990, Ministry of Health‐Republic of Indonesia, 2012). Nurses are the main health care professionals at the Puskesmas and they carry out most of the national health programs (Assan, Assan, Assan & Smith, 2009; Hennessy, Hicks, Hilan & Kawonal, 2006). Therefore, primary health care nurses have a crucial responsibility for managing the delivery of safe and effective health programs in Indonesia (Hennessy et al., 2006).

The context and situation presented above indicate the importance of carrying out a study to assess the attitudes of Indonesian primary health care nurses towards nursing research and the use of research to guide their practice. Such a study will enable us to understand the factors that influence nursing research utilization in Indonesian primary health care settings and facilitate Indonesian nurses to participate in research. This research requires a reliable and valid instrument to measure the variable of interest—attitudes towards research and research utilization—in the context of Indonesian primary health care settings.

## 3 The study

### 3.1 Aim

The aim of this research was to translate, adapt and test the psychometric properties of ATRAD‐N in Indonesian primary health care nurses. The objectives of the study were to:

1. translate a previously developed questionnaire, ATRAD‐N, from the source language (English) to the target language (Indonesian)
2. evaluate and adapt the questionnaire in terms of items, instruction for administration and scoring rules
3. estimate the content and construct validity of the translated questionnaire and its homogeneity reliability

### 3.2 Method

The translation process was conducted systematically by applying the forward and back‐translation method (Beaton, Bombardier, Guillemin & Ferraz, 2000; Gudmundsson, 2009; Sousa & Rojjanasrirat, 2011), involving two native Indonesian speakers fluent in English and a bilingual translator who blindly back‐translated the preliminary initial instrument into English.

Six experts with backgrounds in community health nursing from various universities in Indonesia reviewed the instrument based on local information, context and the culture where the instrument was to be applied. Items 36–39 were deleted because they are not relevant to Indonesian settings. The outcome of the adaptation process was the development of a final Indonesian version of the instrument (Indonesian ATRAD‐N). Ten new items relating to biographical details were generated to assess basic information of the respondents.

**Figure 1:**
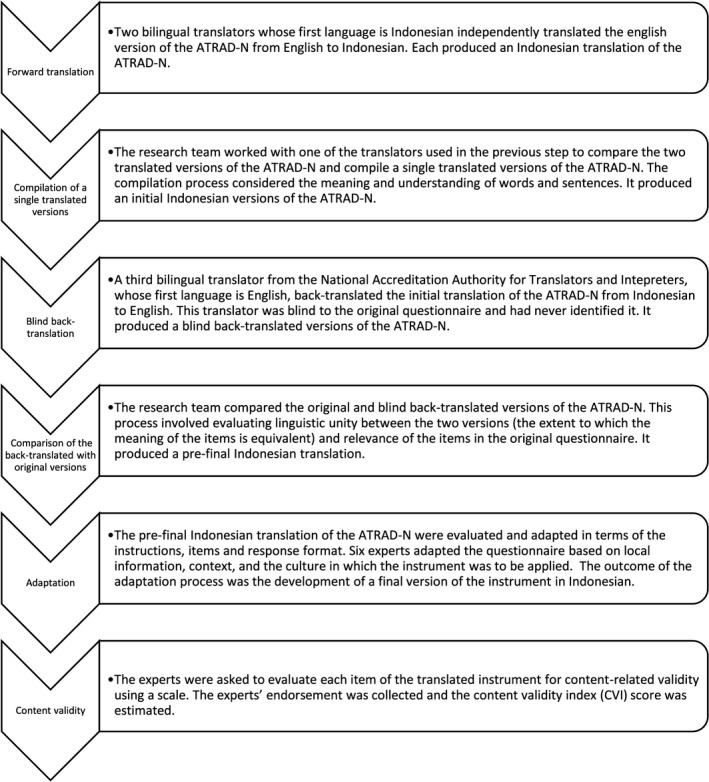
Translation, adaptation and content validity process of the Indonesian ATRAD‐N instrument

The Lynn method of content validity scale was used to quantify the indicators of content appropriateness and relevance provided by the experts in this study (Devon et al., 2007; Lynn, 1986). The experts’ endorsement was collected and the Content Validity Index (CVI) score was estimated for individual scale items and the entire scale. For a panel of six experts, the level of endorsement required to retain an item based on the proportion of the experts would be a minimum of .83, at the .05 level of significance (Lynn, 1986; Wynd, Schmidt & Schaefer, 2003). Figure 1 illustrates the translation, adaptation and content validity process applied to the Indonesian ATRAD‐N.

The process of psychometric testing included validity and reliability tests. Construct validity testing was conducted using factor analysis and homogeneity reliability testing was conducted using Cronbach's alpha coefficient (Devon et al., 2007; Gillespie & Chaboyer, 2013). Cronbach's alpha was calculated for individual factors and the entire scale.

The Indonesian ATRAD‐N included both positively and negatively worded statements. The negatively phrased items were reverse scored in data analysis. Returned questionnaires with more than 10% unanswered items were excluded. For questionnaires with less than 10% of items unanswered, missing data were derived using mean estimation. All data were gathered using hard‐copy questionnaires. The data were coded and entered into a Microsoft Excel spread sheet and then exported into the Statistical Package for the Social Sciences (SPSS) Version 20.0 for data cleaning, reverse scoring and further analysis.

Exploratory factor analysis was performed using Principal Component Analysis (PCA). The factors obtained were then rotated using Direct Oblimin rotation (Pallant, 2011). The criteria for the significance of factor loadings was set at .55 based on the sample size of 92 respondents (Hair, Anderson, Tatham & Black, 1995). Criteria used in determining factor extraction included targets for the eigenvalue >1 rule, cumulative percentage of variance 50–60%, scree test and parallel analysis (Hair et al., 1995; Kootstra, 2004; Pallant, 2011; Pett, Lackey & Sullivan, 2003; Williams, Brown & Onsman, 2010).

The internal consistency of the instrument was measured using Cronbach's alpha coefficient, comparing each item in the scale with all other items. A minimum score of .70 was set to ensure adequate reliability (Gillespie & Chaboyer, 2013). The demographic data were statistically analysed and tested to compare the mean scores in each and total factors using independent sample t‐tests. The correlation between factors derived from the factor analysis was measured using the Spearman rank‐order correlation.

### 3.3 Participants

The subjects of the psychometric tests were recruited using a non‐probability sampling method, from primary health care nurses working in 34 public health centres in the city of Banjarbaru and Banjarmasin, South Kalimantan, Indonesia. In performing factor analysis, Devon et al. (2007) suggested five subjects per item of the questionnaire. With 34 items in the questionnaire, we expected to have 170 respondents. However, 25 centres declined to take part in this study due to heavy workloads or, in some cases, because they felt this study would not benefit them. A total of 92 nurses were completed the instrument in 2013.

### 3.4 Instrument

We critically examined available tools and selected ATRAD‐N based on its adaptation for use in primary health care settings and its published validity and reliability to measure nurses’ attitudes towards research and research utilization. The current ATRAD‐N consists of 35 items including a Likert‐type (1–5) scale with responses ranging from “do not agree at all” (1) ‐ “agree to a very great extent” (5) and four items including a rating type scale. A higher total score indicates a more positive attitude towards research and research utilization.

### 3.5 Ethical considerations

Ethics approval was obtained from the Human Research Ethics Committee at The University of Adelaide (project number HS‐2013‐041). Permission for the translation, adaptation and psychometric testing of the questionnaire was obtained from one of the originators of the questionnaire (Monica E. Björkström from Karlstad University, Sweden). The information sheet was given to potential participants prior to the study being conducted. The respondent's willingness to complete the instrument was taken as an indication of their consent.

## 4 Results

### 4.1 Characteristics of the respondents

A demographic profile of the 92 respondents is presented in Table 1. Most respondents (69.6%) were female and most (78.2%) were aged between 20–40 years old. They were predominantly (71.7%) educated at the diploma level. Almost 59% of the respondents also had research‐related education. The means of the overall response score for each item in the questionnaire (following reverse scoring) ranged from 2.1‐4.3. A higher score indicated a more positive attitude towards research and research utilization.

**Table 1:** Demographic data regarding respondents

Variable	*N*	%
Gender		
Male	28	30.4
Female	64	69.6
Age (years)		
20–30	37	40.2
31–40	35	38.0
41–50	16	17.4
>50	4	4.3
Education level		
Vocational school	11	12.0
Diploma degree	66	71.7
Bachelor degree	15	16.3
Length of working experience (years)		
<1	3	3.3
2–5	32	34.8
6–10	27	29.3
11–15	13	14.1
>15	17	18.5
Access to the Internet at work		
Yes	16	17.4
No	76	82.6
Research experience		
Yes	55	59.8
No	37	40.2
Research‐related education		
Yes	54	58.7
No	38	41.3

### 4.2 Results of content validity process

The instrument was designated as valid by the experts with a CVI of .97 for the entire scale. One item, “nursing education programs are too research based” (CVI = .67) was dropped because it did not achieve the .83 level of endorsement required to establish content validity. Thus, the final version of the instrument in Indonesian consisted of 34 items from the ATARD‐N and 10 items relating to biographical details of respondents.

### 4.3 Results of factor analysis

The 34 items in the questionnaire were subjected to PCA. Prior to performing factor analysis, the data were assessed using two statistical measures: Bartlett's test of sphericity (Bartlett, 1954) and the Kaiser–Meyer–Olkin (KMO) measure of sampling adequacy (Kaiser, 1974) generated by SPSS. Bartlett's test reached a significance point (χ^2^ = 1766.7, *df* = 561, *p* < .0001), which indicated that the correlation matrix was not an identity matrix. The KMO value was .759, which exceeds the recommended value of .6 and meets the “middling” criterion (Kaiser, 1974) which judges our sample size as sufficient to perform factor analysis.

The factor analysis used three iterative analyses. The criteria for the significance of factor loadings was set at .55 based on the sample size of 92 respondents (Hair et al., 1995). The first iteration of the PCA identified the presence of 10 components with eigenvalues >1 explaining 73% of the cumulative percentage of variance. After Direct Oblimin rotation, the pattern matrix showed 10 factors and five components on which only one item loaded (items 3, 7, 8, 9 and 10). Several items (*n *=* *15) did not load on any factors. The results of the parallel analysis indicated only five components with eigenvalues greater than the criterion value for a randomly generated data matrix of 34 items with 100 respondents. It was decided to retain five components for further investigation.

The second iteration of the PCA was run by adding commands to force items loading onto five components. The pattern matrix showed five components with two to six items loading on each component, explaining 55.2% of the cumulative percentage of variance. However, 12 items did not load on any of the components. Each of these was evaluated for possible deletion. It was decided that item 3 “in the nursing area too much is written and there is too much talk about research and development” could be deleted due to its low communality index (.273). The third iteration of the PCA was performed using 33 items and extracted five components. The cumulative percentage of variance was 56.5% with components 1, 2, 3, 4 and 5 contributing 30.35, 8.23, 6.39, 6.00 and 5.39%, respectively.

Table 2 presents the pattern matrix and the structure matrix showing all loadings, including the communality index for each item. The final solution of five components extracted were further labelled as Factor 1 “Participation and utilization of nursing research”, Factor 2 “Nursing professional development”, Factor 3 “Language of nursing research”, Factor 4 “Developing capacity of nurses” and Factor 5 “Need of nursing research”.

**Table 2:** Pattern matrix, structure matrix and communalities during the final iteration

Items (*n *= 33)	Pattern coefficient component					Structure coefficient component					Communalities
	1	2	3	4	5	1	2	3	4	5	
28. I do not bother to find out about research results	**.750**	−.132	.087	.027	.115	.**703**	.190	.038	−.116	−.224	.527
31. It is not meaningful to devote oneself to research in nursing	.**746**	.125	.036	−.011	−.129	.**814**	.227	−.018	−.201	−.471	.697
26. It is unrealistic to believe one can apply research results to practical nursing	.**734**	.172	−.074	.121	−.040	.**749**	.249	−.116	−.061	−.364	.608
33. Introducing changes and testing new ideas is very important in the nursing profession	.**734**	−.202	.036	−.080	−.101	.**771**	−.102	−.026	−.242	−.393	.644
26. Participating in research should be part of the nurse's job	.**732**	.097	−.062	.054	−.010	.**739**	.171	−.110	−.118	−.331	.561
13. I am keen to participate in international scientific conferences	.**673**	−.099	−.167	−.058	−.045	.**706**	−.019	−.221	−.219	−.328	.541
30. It is self‐evident that the nursing profession should be based on scientific and reliable experience	.**594**	.069	−.271	−.057	.004	.**632**	.131	−.317	−.211	−.275	.481
32. Nurses should take the time to read research reports	.**558**	.137	.225	−.107	−.158	.**647**	.232	.177	−.246	−.438	.528
29. Students on the nursing program are/should be a resource in the workplace to stimulate the development of nursing	.**551**	−.067	−.147	−.287	−.070	.**646**	.019	−.211	−.427	−.346	.532
24. Nursing research is essential for me in my development as a professional nurse	.423	.285	.171	−.418	−.192	.**614**	.390	.109	−.548	−.497	.714
4. I think it is interesting to read scientific articles about nursing care	.367	−.101	.237	−.251	−.295	.520	.007	.187	−.359	−.477	.465
22. Taking part in research does not lead to greater professional skill as a nurse	−.031	.**789**	−.182	−.147	−.191	.178	.**826**	−.185	−.235	−.345	.780
16. Nursing research does not raise the status of the nursing profession	.164	.**774**	−.192	−.080	.050	.255	.**785**	−.201	−.168	−.174	.693
21. We should have more nurses in clinical work with a PhD/postgraduate education	.081	.**687**	−.173	.004	−.028	.176	.**698**	−.171	−.073	−.185	.528
34. I think the questions in this questionnaire are important	.101	.**638**	.244	−.054	.180	.085	.**622**	.241	−.061	.017	.470
9. The language of scientific articles is much too complex for me	.076	.295	**−**.**650**	.192	−.209	.202	.322	−.**639**	.069	−.265	.595
20. The language used in nursing research is too complex	.375	.097	**−**.**632**	.064	−.199	.502	.161	−.**654**	−.109	−.371	.684
19. My position as a nurse is sufficiently strong to be able to influence nursing without having knowledge of research	.450	−.044	.461	.059	−.199	.484	.040	.431	−.035	−.369	.486
10. It is not meaningful to get involved in development work in nursing	.258	−.013	.123	**−**.**725**	−.029	.418	.063	.046	**−**.**776**	−.266	.683
11. Being involved in development work in nursing should be part of the nurse's job	.188	.104	.158	**−**.**659**	−.201	.417	.200	.091	**−**.**730**	−.416	.678
2. Participating in development work in nursing does not benefit nursing skills	−.178	.233	.057	**−**.**562**	−.223	.059	.288	.025	−.573	−.288	.444
18. A PhD for nurses should be a prerequisite for certain senior positions in nursing	.322	−.013	.136	.487	−.296	.332	.046	.149	.376	−.343	.411
16. Lecturers on the nursing should be a nursing development resource in the workplace to stimulate the development of nursing	.172	−.029	−.337	−.466	−.283	.415	.063	−.390	**−**.**580**	−.438	.596
12. We do not need nursing scientists to develop patient care, the practice nurses can do that themselves	−.066	−.054	−.123	.073	**−**.**753**	.243	.067	−.120	−.054	**−**.**703**	.520
5. The nursing profession does not require research based knowledge to the same extent as the medical profession	.172	−.055	−.016	.214	**−**.**654**	.400	.066	−.018	.061	**−**.**680**	.523
14. Nursing research complicates the ordinary work of nursing	.045	.221	−.170	−.201	**−**.**608**	.384	.344	−.192	−.346	**−**.**704**	.624
1. As a nurse you must be able to read literature in English	−.023	−.116	−.053	−.201	**−**.**607**	.272	.000	−.075	−.302	**−**.**613**	.430
23. The results of nursing research must be disseminated better to nurses in their work	.117	−.017	.104	−.177	**−**.**581**	.395	.110	.076	−.297	**−**.**659**	.487
6. Nursing science and nursing research describes nursing care and makes it visible	.023	.178	−.006	−.226	**−**.**570**	.336	.295	−.029	−.345	**−**.**652**	.511
7. The nursing profession is a practical profession and does not have to include research	.205	.111	.313	.029	**−**.**562**	.428	.234	.297	−.098	**−**.**661**	.575
18. Further training in research and research‐based studies is not important for the future	.151	.081	−.098	−.099	**−**.**553**	.425	.200	−.121	−.244	**−**.**651**	.476
27. Proficiency in nursing is primarily attained through long practical experience	−.370	.367	.339	.361	−.491	−.223	.398	.393	.358	−.330	.653
8. Research literature on nursing should be available at the workplace	.292	−.336	−.254	−.140	−.365	.462	−.235	−.293	−.269	−.458	.493

Major loadings for each item are in bold.

To interpret these components, Direct Oblimin rotation was performed. The rotated solution revealed the presence of a simpler solution and found seven items that did not load on any of the components. These seven items were consequently removed from further testing of the resulting 26 item instrument. All unloaded items are listed in Table 3.

**Table 3:** Unloaded items with factor loadings <.55 from the final iteration

Items (*n* = 7)	Factor loadings
Nursing research is essential for me in my development as a professional nurse	.423
I think it is interesting to read scientific articles about nursing care	.367
My position as a nurse is sufficiently strong to be able to influence nursing without having knowledge of research	.461
A PhD for nurses should be a prerequisite for certain senior positions in nursing	.487
Lecturers on the nursing education program are/should be a resource in the workplace to stimulate the development of nursing	−.466
Proficiency in nursing is primarily attained through long practical experience	−.491
Research literature on nursing should be available at the workplace	−.365

### 4.4 Results of internal consistency (homogeneity reliability test)

The homogeneity reliability of the instrument's 26 items was measured using Cronbach's alpha coefficient. Bjorkstrom and Hamrin (Bjorkstrom & Hamrin, 2001) showed that the ATRAD‐N questionnaire has a good internal consistency, with a Cronbach's alpha coefficient of .940. In this study, the overall Cronbach's alpha coefficient of the 26 item instrument was .902. Considering that this study extracted different factors than that of Bjorkstrom and Hamrin (2001), it was decided not to compare the Cronbach's alpha coefficient for each factor between the two studies.

The inter‐item correlation matrix values were all positive, indicating that all the items have been correctly reverse scored. Cronbach's alpha for factors ranged from .719 (Factor 4: developing capacity of nurses) ‐ .884 (Factor 1: participation and utilization of nursing research). Two items had higher Cronbach's alpha values than the factor values: “I think the questions in this questionnaire are important” (α = .800) and “Participating in development work in nursing does not benefit nursing skills” (α = .792). Table 4 reports the Cronbach's alpha coefficients for the entire scale and individual factors. The values of deleted items are also included, together with the mean and standard deviation of the value for each item.

**Table 4:** The Cronbach's alpha coefficients for the entire scale and individual factors

Factors and items	α	α if item deleted	(+)/(−)	Mean	*SD*	*n*
Factor 1 “participation and utilization of nursing research” (nine items)	.884					
I do not bother to find out about research results		.875	(+)	30.8	.7	92
It is not meaningful to devote oneself to research in nursing		.862	(−)	30.8	.6	92
It is unrealistic to believe one can apply research results to practical nursing		.869	(−)	30.8	.6	92
Introducing changes and testing new ideas is very important in the nursing profession		.867	(+)	40.1	.7	92
Participating in research should be part of the nurse's job		.870	(+)	30.7	.8	92
I am keen to participate in international scientific conferences		.870	(+)	30.8	.8	92
It is self‐evident that the nursing profession should be based on scientific and reliable experience		.882	(+)	30.8	.9	92
Nurses should take the time to read research reports		.876	(+)	30.9	.6	92
Students on the nursing programs are/should be a resource in the workplace to stimulate the development of nursing		.872	(+)	30.9	.8	92
Factor 2 “nursing professional development” (four items)	.782					
Taking part in research does not lead to greater professional skill as a nurse		.662	(−)	30.5	10.0	92
Nursing research does not raise the status of the nursing profession		.708	(−)	30.6	10.1	92
We should have more nurses in clinical work with a PhD/postgraduate education		.717	(+)	30.0	10.1	92
I think the questions in this questionnaire are important		.800	(+)	30.8	.7	92
Factor 3 “language of nursing research” (two items)	.821					
The language of scientific articles is much too complex for me		.696^a^	(−)	30.2	.9	92
The language used in nursing research is too complex		.696^a^	(−)	30.2	.9	92
Factor 4 “developing capacity of nurses” (three items)	.719					
It is not meaningful to get involved in development work in nursing		.548	(−)	40.1	.7	92
Being involved in development work in nursing should be part of the nurse's job		.482	(+)	40.0	.6	92
Participating in development work in nursing does not benefit nursing skills		.792	(−)	40.2	.6	92
Factor 5 “need of nursing research” (eight items)	.828					
We do not need nurse scientists to develop patient care, the practice nurses can do that themselves		.818	(−)	30.5	.9	92
The nursing profession does not require research‐based knowledge to the same extent as the medical profession		.806	(−)	30.9	.9	92
Nursing research complicates the ordinary work of nursing		.798	(−)	30.7	.7	92
As a nurse, you must be able to read literature in English		.814	(+)	30.9	.7	92
The results of nursing research must be disseminated better to nurses in their work		.805	(+)	40.3	.6	92
Nursing science and nursing research describes nursing care and makes it visible		.808	(+)	40.4	.6	92
The nursing profession is a practical profession and does not have to include research		.806	(−)	40.1	.7	92
Further training in research and research‐based studies is not important for the future		.812	(−)	40.1	.5	92
Overall Cronbach's alpha if factor is ignored	.902					

a Mean inter‐item correlation for the item.

### 4.5 Results of bivariate analysis

A series of independent‐sample *t* tests was conducted to compare the questionnaire scores for several dichotomous socio‐demographic factors. Table 5 displays total and each factor scores split by level of education, length of working experience, Internet access, research education and research experience. The total scores could be between 26 and 130 and the respondents’ scores varied between 64 and 127. The mean value was 99.15 (*SD* 10.74).

**Table 5:** Independent sample *t* test scores

	Factor 1 “participation and utilisation of nursing research”^b^				Factor 2 “nursing professional development”^c^				Factor 3 “language of nursing research”^d^				Factor 4 “developing capacity of nurses”^e^				Factors 5 “need of nursing research”^f^				Total scores^g^			
	*n*	Mean	*SD*	*p*	*n*	Mean	*SD*	*p*	*n*	Mean	*SD*	*p*	*n*	Mean	*SD*	*p*	*n*	Mean	*SD*	*p*	*n*	Mean	*SD*	*p*
Level of education																								
Non‐university	77	33.9	4.4	.00^a^	77	13.9	2.7	.97	77	6.2	1.6	.07	77	12.2	1.5	.31	77	31.	3.7	.00^a^	77	97.7	10.0	.00^a^
University	15	37.9	4.6		15	13.9	4.8		15	7.1	1.8		15	12.7	1.6		15	35.0	3.1		15	106.6	117	
Number of years working																								
0–10	35	33.9	4.5	.34	35	13.5	2.8	.20	35	6.9	1.5	.02^a^	35	12.3	1.6	.96	35	32.2	3.4	.69	35	98.8	9.9	.79
>10	57	34.9	4.8		57	14.3	3.2		57	6.1	1.7		57	12.3	1.5		57	31.8	4.1		57	99.4	11.3	
Internet access at work																								
Yes	16	36.7	2.4	.00^a^	16	15.0	1.9	1.43	16	6.8	1.8	.31	16	13.1	1.4	.03^a^	16	33..4	3.6	.09	16	104.9	7.3	.02^a^
No	76	34.1	4.9		76	13.8	3.2		76	6.3	1.6		76	12.1	1.5		76	31.7	3.9		76	97.9	10.9	
Research education																								
Yes	55	34.6	5.5	.97	55	13.8	3.5	.54	55	6.5	1.6	.32	55	11.9	1.7	.01^a^	55	31.8	3.9	.54	55	98.6	12.5	.53
No	37	34.5	3.0		37	14.2	2.4		37	6.2	1.6		37	12.8	1.00		37	32.3	3.8		37	99.9	7.6	
Research																								
Yes	54	34.9	5.4	.29	54	14.3	3.2	.24	54	6.80	1.45	.00^a^	54	12.3	1.6	.83	54	32.2	4.2	.56	54	100.5	12.1	.11
No	38	33.9	3.2		38	13.5	2.8		38	5.76	1.72		38	.2.3	1.5		38	31.7	3.4		38	97.2	7.9	

a Mean scores differ between the two groups *(p < *.05).

b ltem range = 9–45.

c Item range = 4–20.

d Item range = 2–10.

e Item range = 3–15.

f Item range = 8–40.

g Item range = 26–130.

Two socio‐demographic factors had a significant difference in mean total factor scores: level of education and access to Internet in the workplace. Nurses who were educated at university level had a higher mean value than those who were educated at non‐university level (*p* = .003). Likewise, nurses who had access to the Internet had a higher mean value than those with no Internet access (*p* = .017).

Further analysis was conducted to describe the strength and direction of the linear relationships between the factors using Spearman rank‐order correlation coefficients (Table 6). There was a strong, positive correlation between total factors and each of Factors 1, 2, 3, 4 and 5 (*r* = .800, .631, .554, .526, .840 respectively, *n* = 92, *p* < .0001) with positive attitudes towards nursing research and development being associated with positive attitudes towards participation and utilization of nursing research, nursing professional development, language of nursing research, developing capacity of nurses and need of nursing research.

**Table 6:** Spearman rank‐order correlation coefficient among total factor and individual factors

	Total factors	Total Factor 1	Total Factor 2	Total Factor 3	Total Factor 4	Total Factor 5
		“Participation and utilization of nursing research	“Nursing professional development^”^	“Language of nursing research”	“Developing capacity of nurses”	“Need of nursing research”
Total factors	*–*	.800^a^	.631^a^	.554^a^	.526^a^	.840^a^
Total Factor 1		*–*	.407^a^	.365^a^	.402^a^	.569^a^
Total Factor 2			*–*	.335^a^	.247^b^	.348^a^
Total Factor 3				*–*	.123	.336^a^
Total Factor 4					*–*	.498^a^
Total Factor 5						*–*

a *p < *.001 (2‐tailed).

b *p *< .05(2‐tailed).

## 5 Discussion

Several studies have focused on assessing instruments designed to measure and assess research utilization in practice and individual factors associated with research utilization (Estabrooks & Wallin, 2004; Frasure, 2008; Squires et al., 2008, 2011). This study contributed to the development of a valid and reliable instrument to measure nursing research and research utilization in developing country.

In the first stage, the original English version of the ATRAD‐N was translated into Indonesian. The guidelines developed by Beaton et al. (2000), Gudmundsson (2009) and Sousa and Rojjanasrirat (2011) was combined for cross‐cultural adaptation of a self‐report instrument to achieve a quality translation. Although Sousa and Rojjanasrirat (2011) stress the importance of using translators with knowledge of health care terminology, this was not possible in the current study due to limited translation facilities with this particular specifications. However, no item was found to be difficult to translate as the concepts were not specifically grounded in medical or nursing knowledge. A small number of items had minor semantic and idiomatic discrepancies between the languages, but those items were revised during discussions with the research team.

An important issue to highlight in this discussion is the factor structure of the instrument. The factor structure described by Bjorkstrom and Hamrin (2001), Marshall et al. (2007) and Nilsson Kajermo et al. (2013) is quite different to that extracted during the factor analysis in this current study. Bjorkstrom and Hamrin (2001) extracted a seven‐factor structure, Nilsson Kajermo et al. (2013) a three‐factor structure and Marshall et al. (2007) a two‐factor structure, while this study found a five‐factor structure.

Instead of using a Maximum Likelihood extraction method, we used PCA with Direct Oblimin rotation to replicate the construct validity and find the most psychometrically sound and acceptable approach in this study. Careful consideration was also given to the sample size and correlations among factors when choosing the factor extraction method. It was also necessary to run three iteration factor analyses and to delete one item during those iterative analyses, resulting in a 33‐item scale.

The factor loading cut off of .55 used in this study was higher than those used in previous studies (.32–.40) (Bjorkstrom & Hamrin, 2001; Marshall et al., 2007; Nilsson Kajermo et al., 2013). The higher factor loading cut off was necessary to maintain a strict power level of 80% and .05 significance with the sample size of 92 respondents. This significance level for the interpretation of factor loadings was determined following the approach outlined by Hair anderson and helped to ensure the validity of our findings despite a lower than anticipated sample size (Hair et al., 1995).

Seven items did not load on any of the extracted factors because their factor loadings were <.55. It could be argued that those unloaded items were not having an adequate explanation in the construct that they failed to represent in the factor structure. Marshall et al. (2007) also encountered problems maintaining construct validity of the ATRAD‐N instrument, due to “abstract constructs” (Marshall et al., 2007). Further, Frasure (2008), in his systematic review, found that the ATRAD‐N did not clearly declare its theoretical framework, which is important to define a construct of the instrument.

Variation in the ATRAD‐N questionnaire construct are apparent in different settings. Marshall et al. (2007) were unable to present adequate factor structure of the instrument because their factor structure accounted for only 28.3% of the total variance. Nilsson Kajermo et al. (2013) found a three‐factor structure that grouped items based on positively and negatively worded items. Perhaps items in the questionnaire are interpreted differently among the varied nursing settings. For example, in the original study by Bjorkstrom and Hamrin (2001), the items “The nursing profession is a practical profession and does not have to include research” and “Further training in research and research‐based studies is not important for the future” loaded to a factor labelled “the profession”, whereas in this study those two items loaded to a factor labelled “need of nursing research”. It is unclear whether these two items were about the profession or nursing research. Further refinement and retesting of this instrument would improve its construct validity.

The Cronbach's alpha coefficient for individual factors of the 26 item instrument ranged from .719 ‐ .884, suggesting good internal consistency of the instrument. None of the items had corrected item‐total correlation scores <.3, indicating that each item correlated well with the total value. However, two items (“I think the questions in this questionnaire are important” (α = .800) and “Participating in development work in nursing does not benefit nursing skills” (α = .792)) had higher individual Cronbach's alpha if item deleted scores than their total factor scores. Removing those items from the instrument may increase the reliability of those factors.

It is interesting to note that the overall Cronbach's alpha score for the questionnaire in this study was >.9, as in the studies of Bjorkstrom and Hamrin (2001) and Nilsson Kajermo et al. (2013). Experts disagree about the ideal score of Cronbach's alpha to determine homogeneity reliability. According to Gillespie and Chaboyer (2013), scores <.7 indicate lack of correlation between items in the instrument and according to Devellis (2003) scores >.9 indicate redundancy of one or more items. Devellis (2003) suggest that an instrument with Cronbach's alpha score >.9 should be shortened because of this strong correlation between items. Some items may be too similar in the instrument used in this study—for example, “The language used in nursing research is too complicated” and “The language of scientific articles are too complex for me”—and it may be better to review the items for redundancy.

Further analysis of biographical information on the respondents in this study assessed basic information regarding Indonesian nurses’ attitudes towards research and research utilization and individual factors associated with it. A strong, positive correlation was found: positive attitudes towards nursing research and development were associated with positive attitudes towards participation and utilization of nursing research, nursing professional development, language of nursing research, developing capacity of nurses and need of nursing research. This fact supported Estabrooks, Floyd, Scott‐Findlay, O'Leary and Gushta (2003a) explanation that beliefs, barriers and facilitators are potential individual elements influencing participation and utilization of nursing research. The current study also found that level of education and access to the Internet significantly influenced nurses’ attitudes towards research and research utilization in Indonesia. However, this extra analysis should be interpreted with caution until it can be confirmed with further studies.

The results of this study indicate a difference in psychometric properties of the ATRAD‐N between the primary language (English) and the target language (Indonesia). The adaptation and psychometric testing of the instrument for use in Indonesian primary health care settings did not mirror previous study findings. In its present form, the Indonesian translation of the ATRAD‐N should be used with some caution as further investigation of the psychometric properties of the instrument is required. Studies with more respondents should be undertaken to better establish the validity and reliability of the instrument.

### 5.1 Limitations

The sample size of this study was small (*n* = 93) given the number of items (*n* = 34) in the translated questionnaire. Even though there is no agreement on an acceptable ratio of cases to variables for factor analysis, a general rule of thumb from the literature is a minimum of five cases for each variable to be analyzed (Devon et al., 2007; Hair et al., 1995; Kootstra, 2004; Williams et al., 2010). However, confidence in our findings is increased by the results of the Bartlett's test of sphericity and the KMO assessment of “middling” for sampling adequacy, which judges our sample size as sufficient to perform factor analysis.

## 6 Conclusion

The respondents for psychometric testing in this study were collected from a different geography, culture and context than previous studies. Following translation, adaptation and psychometric testing, it was found that the ATRAD‐N instrument showed acceptable content validity and homogeneity reliability, but not construct validity in Indonesian settings. Thus, further development, refinement and retesting of the instrument would be essential to produce a psychometrically sound instrument.

## Conflict of interest

No conflict of interest has been declared by the authors.

## Author contributions

Kurnia Rachmawati, Lynette Cusack and Tim Schultz designed the study. Kurnia Rachmawati collected the data. Kurnia Rachmawati and Tim Schultz performed the analyses and interpreted the data. Lynette Cusack and Tim Schultz supervised the study and made critical revisions. Kurnia Rachmawati drafted the manuscript. All authors read and approved the final manuscript.

## References

[nop272-bib-0001] Abdullah, A. , Hort, K. , Abidin, A. Z. , & Amin, F. M. (2012). How much does it cost to achieve coverage targets for primary healthcare services? A costing model from Aceh, Indonesia. The International Journal of Health Planning and Management, 27, 226–245. 22887349 10.1002/hpm.2099

[nop272-bib-0002] Assan, J. K. , Assan, S. K. , Assan, N. , & Smith, L. (2009). Health inequality in resource poor environments and the pursuit of the MDGs: Traditional versus modern healthcare in rural Indonesia. Journal of Health Management, 11, 93–108.

[nop272-bib-0100] Bartlett, M. S . (1954). A note on the multiplying factors for various chi square approximations. Journal of the Royal Statistical Society, 16, 296–298.

[nop272-bib-0003] Beaton, D. E. , Bombardier, C. , Guillemin, F. , & Ferraz, M. B. (2000). Guidelines for the process of cross‐cultural adaptation of self‐report measures. Spine (Phila Pa 1976), 25, 3186–3191. 11124735 10.1097/00007632-200012150-00014

[nop272-bib-0004] Bjorkstrom, M. E. , & Hamrin, E. K. (2001). Swedish nurses’ attitudes towards research and development within nursing. Journal of Advanced Nursing, 34, 706–714. 11380739 10.1046/j.1365-2648.2001.01800.x

[nop272-bib-0005] Department of Health‐Government of Indonesia (1990). Public health center: Manual. Jakarta: Department of Health.

[nop272-bib-0006] Devellis, R. F. (2003). Reliability. Scale development theory and applications (2nd edn). USA: Sage Publication.

[nop272-bib-0007] Devon, H. A. , Block, M. E. , Moyle‐Wright, P. , Ernst, D. M. , Hayden, S. J. , Lazzara, D. J. , Savoy, S. M. , & Kostas‐Polston, E. (2007). A psychometric toolbox for testing validity and reliability. Journal of Nursing Scholarship, 39, 155–164. 17535316 10.1111/j.1547-5069.2007.00161.x

[nop272-bib-0008] Estabrooks, C. A. (2009). Mapping the Research Utilization Field in Nursing. Canadian Journal of Nursing Research, 41, 218–236. 19485054

[nop272-bib-0009] Estabrooks, C. A. , Floyd, J. , Scott‐Findlay, S. , O'Leary, K. , & Gushta, M. (2003a). Individual determinants of research utilization: A systematic review. Journal of Advanced Nursing, 43, 506–520. 12919269 10.1046/j.1365-2648.2003.02748.x

[nop272-bib-0010] Estabrooks, C. A. , & Wallin, L. (2004). Where do we stand on the measurement of research utilization? the 4th Annual Knowledge Utilization Colloquia (KU04). Belfast, Ireland.

[nop272-bib-0011] Estabrooks, C. A. , Wallin, L. , & Milner, M. (2003b). Measuring knowledge utilization in health care. International Journal of Policy Analysis and Evaluation, 1, 3–36.

[nop272-bib-0012] Frasure, J. (2008). Analysis of instruments measuring nurses’ attitudes towards research utilization: A systematic review. Journal of Advanced Nursing, 61, 5–18. 18173733 10.1111/j.1365-2648.2007.04525.x

[nop272-bib-0013] Gillespie, B. , & Chaboyer, W. (2013). Assessing measuring instruments In Schneider Z., Whitehead D., Lobiondo‐Wood G. & Haber J. (Eds.), Nursing and midwifery research: Methods and appraisal for evidence‐based practice (4th edn, pp. 218–233). NSW: Mosby Elsevier.

[nop272-bib-0014] Gudmundsson, E. (2009). Guidelines for translating and adapting psychological instruments. Nordic Psychology, 61, 29–45.

[nop272-bib-0015] Hair, J. F. J. , Anderson, R. E. , Tatham, R. L. , & Black, W. C. (1995). Factor Analysis. Multivariate data analysis: With readings (4th edn). New Jersey: Prentice Hall.

[nop272-bib-0016] Hennessy, D. , Hicks, C. , Hilan, A. , & Kawonal, Y. (2006). The training and development needs of nurses in Indonesia: Paper 3 of 3. Human Resources for Health, 4, 10. 16630363 10.1186/1478-4491-4-10 PMC1524804

[nop272-bib-0017] Indonesian National Nurses Association (2005). Indonesian nurses competency standard [Online]. Available from: http://www.inna-ppni.or.id/innappni/mntop-standar-kompetensi.html [last accessed 20 March 2013].

[nop272-bib-0101] Kaiser, H. (1974). An index of factorial simplicity. Psychometrika, 39, 31–36.

[nop272-bib-0018] Kootstra, G. J. (2004). Exploratory Factor Analysis: Theory and Application [Online]. Available from: http://www.let.rug.nl/%7Enerbonne/teach/rema-stats-meth-seminar/Factor-Analysis-Kootstra-04.PDF [last accessed 2 July 2013].

[nop272-bib-0019] Lynn, M. R. (1986). Determination and quantification of content validity. Nursing Research, 35, 382–385. 3640358

[nop272-bib-0020] Marshall, A. P. , Fisher, M. J. , Brammer, J. , Eustace, P. , Grech, C. , Jones, B. , & Kelly, M. (2007). Assessing psychometric properties of scales: A case study. Journal of Advanced Nursing, 59, 398–406. 17608684 10.1111/j.1365-2648.2007.04316.x

[nop272-bib-0021] McKenna, H. , Ashton, S. , & Keeney, S. (2004). Barriers to evidence based practice in primary care: A review of the literature. International Journal of Nursing Studies, 41, 369–378. 15050848 10.1016/j.ijnurstu.2003.10.008

[nop272-bib-0022] Mehrdad, N. , Salsali, M. , & Kazemnejad, A. (2008). Iranian nurses’ attitudes toward research utilisation. Journal of Research in Nursing, 13, 53–65.

[nop272-bib-0023] Ministry of Health‐Republic of Indonesia (2012). National Health System 2012. Jakarta: Author.

[nop272-bib-0024] Nilsson Kajermo, K. , Alinaghizadeh, H. , Falk, U. , Wandell, P. , & Tornkvist, L. (2013). Psychometric evaluation of a questionnaire and primary healthcare nurses’ attitudes towards research and use of research findings. Scandinavian Journal of Caring Sciences, 20, 12037. 10.1111/scs.12037 23517064

[nop272-bib-0025] Oh, E. G. (2008). Research activities and perceptions of barriers to research utilization among critical care nurses in Korea. Intensive and Critical Care Nursing, 24, 314–322. 18243707 10.1016/j.iccn.2007.12.001

[nop272-bib-0026] Olade, R. A. (2004). Evidence‐based practice and research utilization activities among rural nurses. Journal of Nursing Scholarship, 36, 220–225. 15495490 10.1111/j.1547-5069.2004.04041.x

[nop272-bib-0027] Pallant, J. (2011). Factor Analysis SPSS survival manual: A step by step guide to data analysis using SPSS (4th edn). NSW, Australia: Allen & Unwin.

[nop272-bib-0028] Pearson, A. , & Jordan, Z. (2010). Evidence‐based healthcare in developing countries. International Journal of Evidence‐Based Healthcare, 8, 97–100. 21077397

[nop272-bib-0029] Pett, M. A. , Lackey, N. R. , & Sullivan, J. J. (2003). Extracting the initial factors. Making sense of factor analysis: The use of factor analysis for instrument development in health care research. Thousand Oaks, CA: Sage Publication.

[nop272-bib-0030] Schneider, Z. (2013). The significance of nursing and midwifery research In Schneider Z., Whitehead D., Lobiondo‐Wood G. & Haber J. (Eds.) Nursing and Midwifery Research: Methods and appraisal for evidence‐based practice (4th edn, pp. 1–15). NSW: Mosby Elsevier.

[nop272-bib-0031] Sousa, V. D. , & Rojjanasrirat, W. (2011). Translation, adaptation and validation of instruments or scales for use in cross‐cultural health care research: A clear and user‐friendly guideline. Journal of Evaluation in Clinical Practice, 17, 268–274. 20874835 10.1111/j.1365-2753.2010.01434.x

[nop272-bib-0032] Squires, J. , Adachi, A. , & Estabrooks, C. (2008). Developing a Valid and Reliable Measure of Research Utilization Technical Report. Edmonton: Faculty of Nursing, University of Alberta.

[nop272-bib-0033] Squires, J. , Estabrooks, C. , O'Rourke, H. , Gustavsson, P. , Newburn‐Cook, C. , & Wallin, L. (2011). A systematic review of the psychometric properties of self‐report research utilization measures used in healthcare. Implementation Science, 6, 83. 21794144 10.1186/1748-5908-6-83 PMC3169486

[nop272-bib-0034] Tsai, S.‐L. (2000). Nurses’ participation and utilization of research in the Republic of China. International Journal of Nursing Studies, 37, 435–444. 10785534 10.1016/s0020-7489(00)00023-7

[nop272-bib-0035] Williams, B. , Brown, T. , & Onsman, A. (2010). Exploratory factor analysis: A five‐step guide for novices. Australasian Journal of Paramedicine, 8, 1–10.

[nop272-bib-0036] Wynd, C. A. , Schmidt, B. , & Schaefer, M. A. (2003). Two quantitative approaches for estimating content validity. Western Journal of Nursing Research, 25, 508–518. 12955968 10.1177/0193945903252998

